# Two-color diffuse *in vivo* flow cytometer

**DOI:** 10.1117/1.JBO.29.6.065003

**Published:** 2024-05-30

**Authors:** Amber L. Williams, Augustino V. Scorzo, Rendall R. Strawbridge, Scott C. Davis, Mark Niedre

**Affiliations:** a Northeastern University, Department of Bioengineering, Boston, Massachusetts, United States; b Dartmouth College, Thayer School of Engineering, Hanover, New Hampshire, United States

**Keywords:** optical devices, fluorescence, circulating tumor cells, circulating tumor cell clusters

## Abstract

**Significance:**

Hematogenous metastasis is mediated by circulating tumor cells (CTCs) and CTC clusters (CTCCs). We recently developed “diffuse *in vivo* flow cytometry” (DiFC) to detect fluorescent protein (FP) expressing CTCs in small animals. Extending DiFC to allow detection of two FPs simultaneously would allow concurrent study of different CTC sub-populations or heterogeneous CTCCs in the same animal.

**Aim:**

The goal of this work was to develop and validate a two-color DiFC system capable of non-invasively detecting circulating cells expressing two distinct FPs.

**Approach:**

A DiFC instrument was designed and built to detect cells expressing either green FP (GFP) or tdTomato. We tested the instrument in tissue-mimicking flow phantoms *in vitro* and in multiple myeloma bearing mice *in vivo*.

**Results:**

In phantoms, we could accurately differentiate GFP+ and tdTomato+ CTCs and CTCCs. In tumor-bearing mice, CTC numbers expressing both FPs increased during disease. Most CTCCs (86.5%) expressed single FPs with the remainder both FPs. These data were supported by whole-body hyperspectral fluorescence cryo-imaging of the mice.

**Conclusions:**

We showed that two-color DiFC can detect two populations of CTCs and CTCCs concurrently. This instrument could allow study of tumor development and response to therapies for different sub-populations in the same animal.

## 1 Introduction

In hematogenous metastasis, circulating tumor cells (CTCs) shed from primary tumors, intravasate into blood vessels, travel through the circulatory system, and may form secondary tumors. Although CTCs are rare (on the order of 1 to 100 CTCs per mL of peripheral blood), their numbers have been shown to be associated with overall patient prognosis and response to treatment.^1^^,^^2^ Multi-cellular CTC clusters (CTCCs) are more rare than single CTCs but are purported to have 50 to 100 times higher metastatic potential.^3^^–^^5^

The primary method of counting and studying CTCs is liquid biopsy in which CTCs are isolated from blood samples.^6^ However, we and others have shown that CTC numbers in small blood samples are often not representative of the entire patient blood volume and may even fail to capture rare CTCs or CTCCs.^7^^–^^11^ Drawing blood samples also makes longitudinal small animal studies difficult, since non-terminal blood draws are typically limited to 200  μL every two weeks without fluid replacement. Therefore, *in vivo* measurements of CTCs offer the ability to sample larger volumes of blood over short and long time periods to study CTC frequency and patterns.

Several optical methods have been developed to detect circulating cells directly in the bloodstream in live animals or in humans, generally termed “*in vivo* flow cytometry” (IVFC).^12^ For example, photoacoustic IVFC relies on the photoacoustic effect for detection of pigmented cell types (such as melanoma)^13^^,^^14^ or cells labeled with absorbing exogenous contrast agents, such as carbon nanotubes.^15^ Other groups have developed and applied confocal fluorescence microscopy-based IVFC instruments for detection of CTCs labeled with organic fluorophores or modified to express fluorescent proteins (FPs).^16^^–^^18^

Our team developed “diffuse *in vivo* flow cytometry” (DiFC) to non-invasively detect and count rare, fluorescently labeled or FP expressing CTCs in small animals using diffuse light.^12^^,^^19^^–^^22^ In contrast to intravital microscopy-based methods, DiFC uses highly scattered light to probe large, relatively deep blood vessels in bulk tissue. Large vessels—for example in the tail or leg of a mouse—carry on the order of 100  μL of blood per minute.^20^ In mice, suitable blood vessels are approximately 1 mm in depth, although we have showed that detection to 2 to 4 mm in tissue is feasible with suitable choice of wavelength and instrument geometry.^23^^,^^24^ Hence, DiFC allows for non-invasive sampling of large peripheral blood volumes and detection of rare cells to, for instance, show that CTC numbers generally increase over the course of disease development in mouse metastasis models, but that they can fluctuate significantly over 24-h periods.^11^^,^^20^^,^^21^

However, the DiFC systems we have developed thus far have been limited to detection of single fluorophores due to specially designed optical fiber bundles with integrated miniaturized filters and lenses that are not easily interchanged.^20^^,^^21^^,^^25^ Given this limitation, we are interested in exploring multiplexed experiments involving monitoring more than one population of cells concurrently with DiFC. This would permit study of CTC shedding in mice with tumors composed of cells of different phenotypes or two different tumors in the same animal.

In this manuscript we report on the design of a two-color DiFC system designed to detect blue-green [green (FP); GFP] and orange (tdTomato) FPs simultaneously. We used the system to monitor CTC numbers in mice inoculated with both GFP and tdTomato expressing multiple myeloma (MM) cells. We demonstrated that the shedding rate of the two populations was uncorrelated. We also validated the ability of two-color DiFC to detect CTCCs containing the two fluorophores both in tissue-mimicking flow phantoms *in vitro* and in MM-bearing mice *in vivo*.

## 2 Materials and Methods

### 2.1 Two-Color DiFC Instrument DiFC

**Fig. 1:**
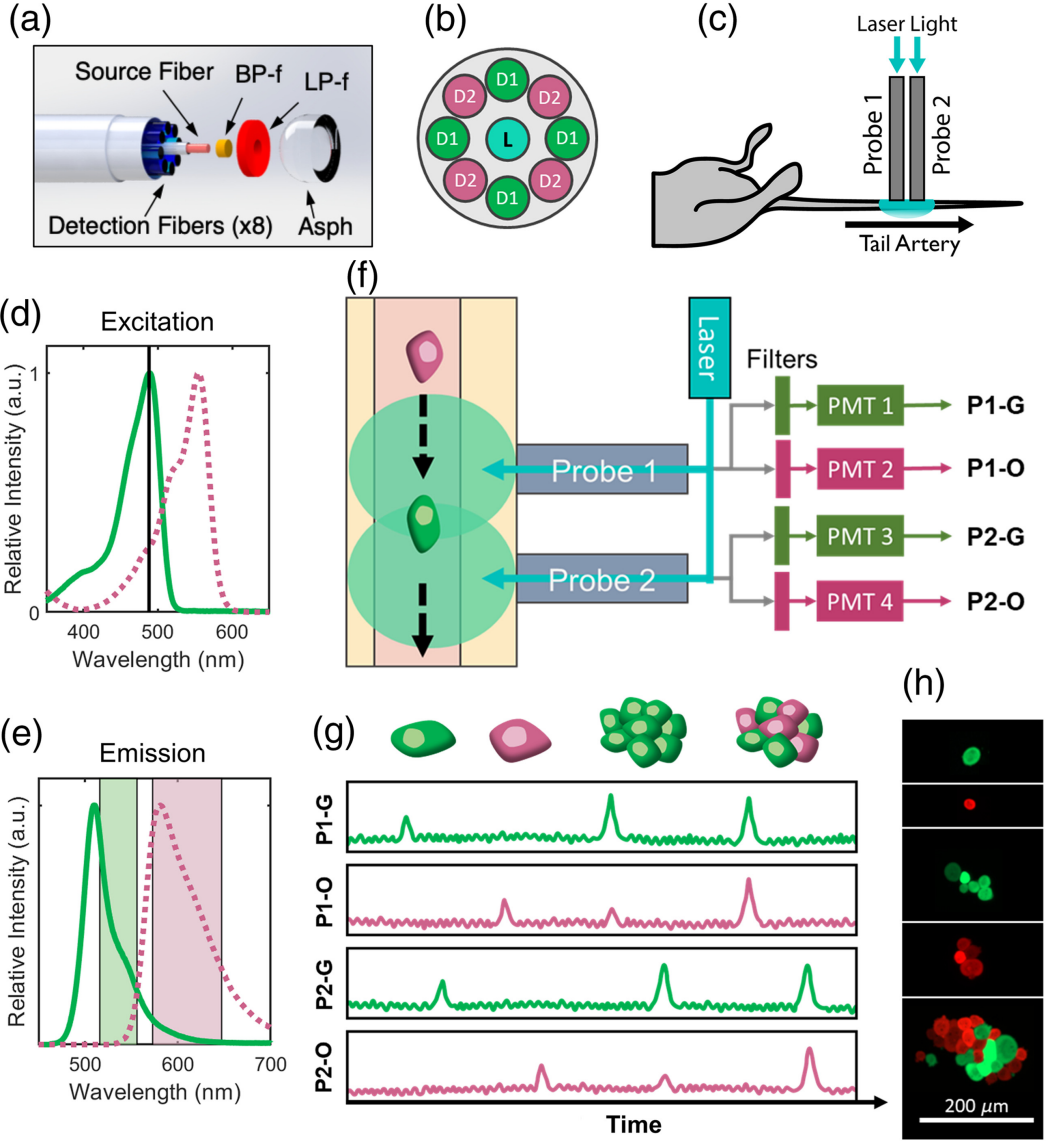
(a) DiFC probe design showing source and detection optical fibers, excitation BP and fluorescence collection LP filters, and aspheric lens tip. Diagram reprinted with permission from Patil et al.^21^ (b) Each two-color DiFC probe contains two sets of four fibers that are interleaved and coupled to two separate detector arms. (c) Two probes are aligned on the mouse tail, approximately above the ventral caudal vascular bundle. (d) The excitation spectra of GFP and tdTomato FP are shown, with a black line indicating the 488 nm laser wavelength. (e) The emission spectra of GFP and tdTomato are shown, along with the two-color DiFC emission filters used for each fluorophore, indicating modest fluorophore “bleed” between channels. (f) FP-expressing cells moving through a blood vessel are excited by laser light. Fluorescent light is collected by the detection fibers and split between two detector arms (filters and PMTs) for each fluorophore. (g) The detected light appears as transient peaks (“spikes”) in light intensity with a time delay between the two probes. In principle, this allows for detection of GFP+ CTCs and clusters, tdTomato+ CTCs and clusters, and two fluorophore multicellular CTC clusters. (h) Example fluorescence microscopy images of each are shown.

The two-color DiFC system uses a similar design to our previously reported GFP-compatible b-DiFC system.^11^^,^^21^^,^^25^ The system uses a 488 nm laser coupled into two specially designed optical fiber probes. Each probe consists of a single source fiber surrounded by a ring of 8 detection fibers [Fig. 1(a)]. The probe tips have internal mounted filters to reduce fiber autofluorescence including a central 488 nm band-pass filter (BP-f) and a ring-shaped 503 nm long-pass filter (LP-f). The eight detection fibers are grouped into two bundles of four which each terminate on an output fiber coupler, emission band-pass filters, a second focusing lens, and photomultiplier tube (PMT). The two sets of detection fibers are interleaved in the probe tip as shown in Fig. 1(b). The tip can then be aligned on the skin surface above a major blood vessel, for instance the ventral tail artery of a mouse [Fig. 1(c)], to excite and detect FP-expressing circulating cells.

GFP and tdTomato were chosen as target FPs. Both tdTomato and GFP can be excited by 488 nm light [tdTomato with lower efficiency than GFP, as shown in Fig. 1(d)], which simplified the instrument construction. To achieve this, one of the two fiber probe outputs is fitted with a 535/50 filter (ET535/50m; Chroma Technology Corporation, Bellows Falls, Vermont, United States) and 536/40 nm filter (FL-004682; IDEX Health and Science, LLC, Rochester, New York, United States) for GFP detection, and the other output with a 610/75 nm filter (ET610/75m; Chroma Technology Corporation) for tdTomato. As we show, the tdTomato and GFP emission spectra are sufficiently separated that the two emission filter sets allow detection of both FPs with minimal inter-channel “bleed” [Fig. 1(e)]. The brightness of tdTomato—up to 2 to 3 times brighter than GFP—allows for detectable emission despite the lower excitation efficiency with the 488 nm laser.^26^

The instrument uses two fiber probes that are placed on the skin surface, approximately above the blood vessel of interest [Fig. 1(c)]. The data from the four PMTs, resulting from the two fiber probes, can be visualized as four channels: probe 1 – green (P1-G), probe 1 – orange (P1-O), P2-G, and P2-O [Fig. 1(f)]. The use of two fiber probes allows us to determine the direction and speed of circulating GFP- and tdTomato-expressing cells. For example, a GFP+ cell moving in a blood vessel beneath probe 1 followed by probe 2 will be detected as peaks in light intensity on channels P1-G and P2-G with a time delay between peaks [Fig. 1(g)]. Similarly, tdTomato+ cells will be detected in channels P1-O and P2-O. This allows us to specifically identify cells moving in the blood vessel of interest as described further in the next section.

### 2.2 Signal Processing and CTC Detection

The signal processing algorithm used for two-color DiFC follows the following steps, which is a modified version of our previously published algorithm:^20^^,^^21^^,^^25^

(i) Subtraction of the signal mean. The DiFC signal includes a non-specific background originating from tissue autofluorescence. To estimate this, we calculate and subtract the median value in a moving 5 s moving window.
(ii) Calculation of the signal noise. Although the background can be removed, the noise in the background is random. We calculate the signal noise post background subtraction in a moving 1-min window.
(iii) Identification of peak candidates. Peak (CTC) candidates are defined as transient maxima with amplitudes equal to or greater than five times the local standard deviation (noise). This gives a minimum signal to noise ratio (SNR) for the peaks of $20\;\log_{10}(5)=13.9\;\mathrm{dB}$.
  Operations i–iii are performed independently for each of the four detector channels P1-G, P1-O, P2-G, and P2-O.
(iv) Candidate peak matching in the forward or reverse directions. To further distinguish CTCs from spurious instrument noise, we impose an additional “matching condition” for peak candidates, wherein peaks must be matched with an appropriate time delay between the two probes in the forward (arterial) or reverse (venous) directions. This peak matching is done separately for green (GFP) and orange (tdTomato) detector pairs. Peaks are matched based on amplitude, width, and time delay between the detectors with respect to the physical probe separation of 3 mm.^20^ Unmatched peaks or simultaneous peaks on all channels are discarded from the analysis. These may arise from instrument noise, motion artifacts, or individual cells in smaller blood vessels, such as the capillary bed. In addition to distinguishing arterial and venous flow, this maintains a very low false alarm rate for DiFC, with zero false directional detections in 6 h.
(v) Identification of two-fluorophore CTC clusters. One goal of the two-color DiFC design is to allow detection of multicellular CTCC composed of both GFP+ and tdTomato+ cells, which we refer to as “two-FP (2FP) CTCCs. As shown diagrammatically in Fig. 1(g), these will be detected as two peaks simultaneously detected in both green and orange channels on one fiber probe. We refer to these peaks as “2λ peaks.” However, because of fluorescence bleed between the detection fluorescence filters [Fig. 1(e)], bright single-FP (1FP) CTCCs may also be detected as 2λ peaks. As such, a signal processing challenge is to distinguish 2FP CTCCs versus 1FP CTCCs with inter-channel bleed, based on the relative amplitudes of the peaks detected between green and orange channels.
  First, 2λ peaks are identified. The green or orange peak with higher amplitude is referred to as the primary peak, and the smaller is the secondary peak. Based on the amplitude of the primary peak, we calculate the largest expected amplitude of a secondary peak that could be attributed to fluorescence bleed between the green and orange channels with the following equation (derivation is provided in the Supplementary Material):
  $$\max(I_{\sec})=I_{\mathrm{pr}}(\frac{\mathrm{TR}*I_{\mathrm{pr}}+5}{I_{\mathrm{pr}}-5}),$$ (1)
  where $I_{\sec}$ and $I_{\mathrm{pr}}$ are the secondary and primary peak amplitudes, respectively. The value TR is estimated by
  $$\mathrm{TR}\approx \frac{\frac{I_{\sec}^{*}}{I_{\mathrm{pr}}^{*}}(I_{\mathrm{pr}}^{*}-5)-5}{I_{\mathrm{pr}}^{*}},$$ (2)
  where $I_{\sec}^{*}$ and $I_{\mathrm{pr}}^{*}$ are known secondary and primary peak amplitudes for 1FP peak detections measured experimentally in flow phantom models *in vitro* as described in Sec. 3.1. The TR value for Eq. (1) is chosen as the lowest value calculated by Eq. (2) such that zero 1FP *in vitro* clusters are detected as two-color.
  If the measured secondary peak amplitude of a 2λ peak is larger than the maximum expected amplitude [Eq. (1)] then the detection is considered a 2FP detection. If the secondary peak does not meet the threshold, then the 2λ peak is considered a 1FP detection.

### 2.3 Cell Lines and CTC Clusters *In Vitro*

#### 2.3.1 Multiple myeloma cells

We used MM.1S cells that had previously been modified to express GFP, firefly luciferase, and neomycin resistance genes (GFP-MM.1S). These cells were originally described by Dr. Rosen at Northwestern University and were previously authenticated by us with an external service (Bio-Synthesis Inc., Lewisville, Texas) to verify their MM.1S lineage.^21^ We also transduced unmodified MM.1S cells (CRL-2974; ATCC, Manassas, Virginia, United States) with a tdTomato and puromycin-resistance lentivirus, LV-EF1α-tdTOMATO-IRES-Puro (SL100323; SignaGen Laboratories, Frederick, Maryland). $6\times 10^{5}$ cells were placed in low protein binding microcentrifuge tubes (022431081; Eppendorf AG, Germany) with RPMI 1640 with no phenol red (11-835-030; Thermo Fisher Scientific Inc, Hampton, NH) containing 10% fetal bovine serum (FBS) and 5  μg/mL polybrene for 10 min at room temperature. Lentivirus was added to the incubating cells with a multiplicity of infection (MOI) of 15 for 2 h at 1200 g and 32°C. The cells were then resuspended in the lentiviral media and additional media and incubated for 24 h before the lentiviral media was removed. The cells were then treated with puromycin to select for the brightest cells.

#### 2.3.2 Breast cancer cells

4T1 cells (CRL-2539; ATCC) were transduced with the tdTomato lentivirus and a GFP lentivirus LV-EF1α-GFP-Puro (SL100269; SignaGen) to create two FP expressing cell lines. Cells were incubated in DMEM (11-995-065; Thermo Fisher Scientific) containing 10% FBS, 5  μg/mL polybrene, and lentivirus with MOI of 10 for 24 h. The lentiviral media was then removed, and the cells were treated with puromycin to select for bright cells.

#### 2.3.3 BC-4T1 CTC clusters grown *in vitro*

To create multicellular clusters, 4T1 cells were incubated for 2 days on tissue culture treated six-well plates. The adherent cells were then washed with phosphate buffer solution (PBS) and suspended in fresh PBS by lifting them from the plates with a cell scraper (08-100-241; Thermo Fisher Scientific). 2FP clusters were formed by co-culturing GFP+ and tdTomato+ cells while 1FP clusters were made with GFP-only or tdTomato-only cultures. No additional drugs or reagents were used to encourage cluster formation. Figure 1(h) shows representative microscope images of *in vitro* single cells and *in vitro*-made clusters.

### 2.4 Optical Flow Phantom Experiments *In Vitro*

To first validate the two-color DiFC system, we used a tissue-mimicking optical flow phantom as we have described previously.^21^ The phantom is a block of scattering plastic that approximately mimics the optical properties and autofluorescence of biological tissue in the visible range. We threaded strands of Tygon tubing (TGY-010-C; Small Parts, Inc., Seattle, Washington, United States) through a through-hole at 0.75 mm depth, mimicking the depth of a blood vessel in a mouse tail. A microsyringe pump is used to pass suspensions of GFP- and tdTomato-expressing cells at final suspension concentrations of approximately $10^{3}$ cells per mL for single cells and $10^{4}$ cells per mL for clusters (which corresponded to approximately $10^{3}$ CTCCs per mL) with a flow speed of 25  μL per minute through the tubing.

### 2.5 Mouse Experiments *In Vivo*

All mice were handled in accordance with Northeastern University’s Institutional Animal Care and Use Committee (IACUC) policies on animal care. Animal experiments were carried out under Northeastern University IACUC protocol #21-0412R. Mice were caged in groups of five or less, and all animals were fed a diet of low fluorescence animal chow (AIN 93M Mature Rodent Diet, Ziegler Feed, East Berlin, Pennsylvania, United States).

We used an MM disseminated xenograft model (MM DXM) (which we have used previously^21^) with an equal mixture of GFP- and tdTomato-expressing MM.1S cells. 8-week-old male severe combined immunodeficient (SCID/Bg) mice (Strain code 250; Charles River Laboratories, Cambridge, Massachusetts, United States) were injected via tail vein with 200  μL of PBS containing $2.5\times 10^{6}$ GFP-MM.1S cells and $2.5\times 10^{6}$ tdTomato-MM.1S cells (N=4). DiFC scanning was performed on each mouse held under inhaled isoflurane when CTCs were expected to enter circulation −28 days.

Additionally, three NOD SCID Gamma (NSG) mice (Strain code 005557; The Jackson Laboratory, Bar Harbor, Maine, United States) were scanned with DiFC for control data.

### 2.6 Whole-Body Hyperspectral Fluorescence Cryo-imaging

To visualize the spatial distribution of GFP and tdTomato expressing cells in the MM DXM model, animals were euthanized and submerged in optimal cutting temperature compound in preparation for hyperspectral fluorescence cryo-imaging. This approach, as described elsewhere,^25^^,^^27^ produces high-resolution 3-D white light and fluorescence images of the entire animal by imaging the frozen specimen during automated serial sectioning. For this study, the specimen was imaged using a white light emitting diode (LED), a 530 nm LED (Mightex, Toronto, Ontario, Canada) with a 550 nm shortpass filter for tdTomato excitation, and a 470 nm LED (Mightex) with a 475 nm shortpass filter for GFP excitation. The resulting RGB and fluorescence image stacks were assembled and rendered using 3D slicer.^28^ In 3D slicer, the GFP and tdTomato tumors were first segmented from the fluorescence image stacks while removing regions of autofluorescence in the stomach and intestines. Next, the overlapping tumor regions expressing both GFP and tdTomato were created by determining the intersection between the GFP and tdTomato segmentations. Finally, the intersecting region was subtracted from both segmentations to generate distinct GFP, tdTomato, and overlapping regions. These segmentations were then rendered into 3-D models for visualization.

## 3 Results

### 3.1 Two-Color DiFC Performance in Phantom

**Fig. 2:**
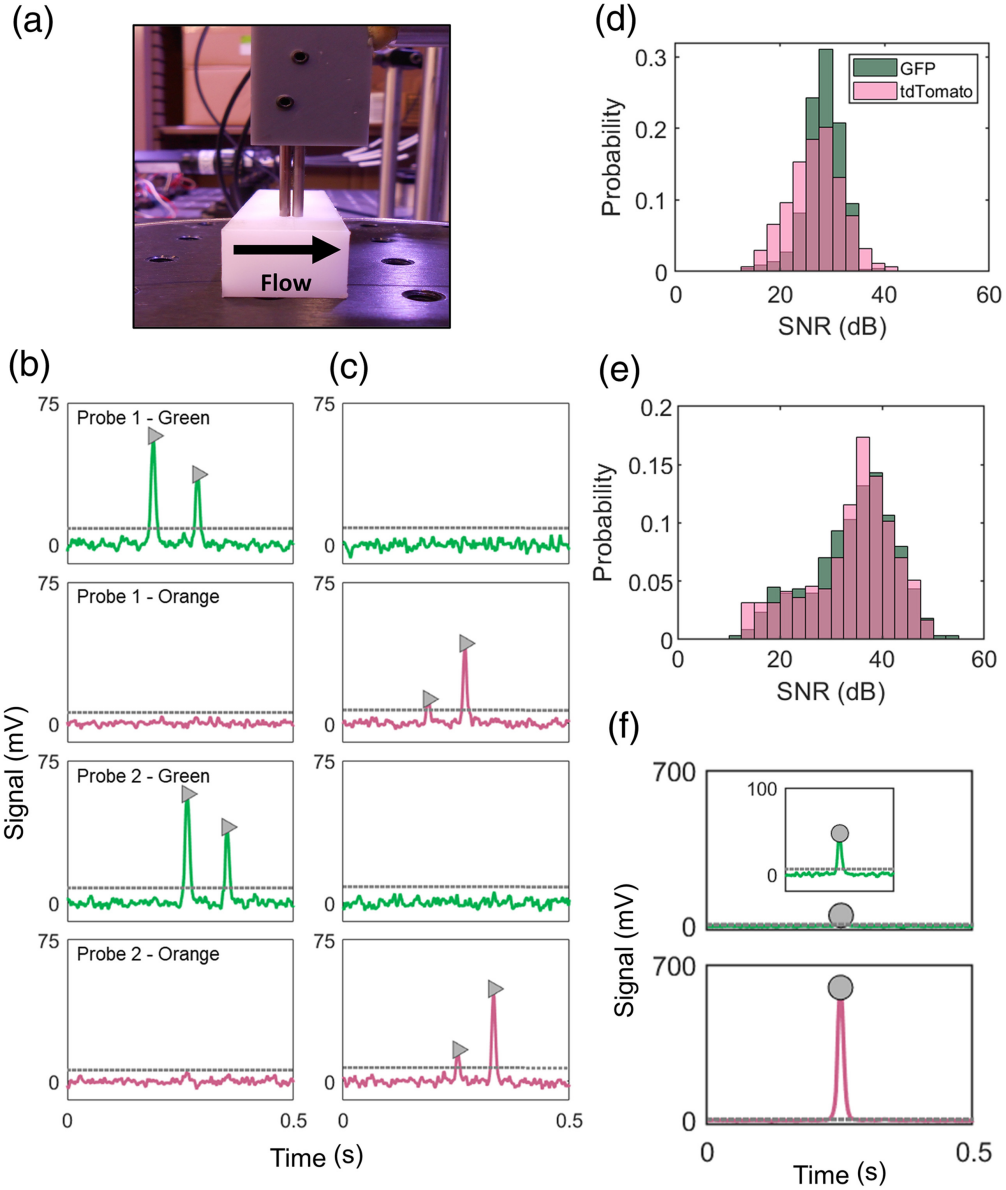
(a) We used an optical flow phantom model for initial *in vitro* validation of two-color DiFC. (b) and (c) Representative DiFC data recorded when suspensions of (b) GFP+ MM cells and (c) tdTomato+ MM cells were passed through the phantom. As shown, GFP+ cells were detected in the green channels, and tdTomato+ cells in the orange channels. (d) and (e) The combination of fluorophore expression and DiFC filters resulted in similar SNRs for both GFP and tdTomato cells. This was the case for both (d) MM and (e) 4T1 cells. (f) Particularly bright cells or 1FP clusters resulted in slight bleed between the two fluorescence channels of either probe. These were distinguished from 2FP clusters by analysis of the relative peak amplitudes as described in Sec. 2.2.

We first performed two-color DiFC in our flow phantom model *in vitro* [Fig. 2(a)] using suspensions of GFP+ and tdTomato+ MM or BC cells. The signals measured from cells expressing either of the FPs are readily distinguishable as they are primarily detected as peaks in either the green [GFP, Fig. 2(b)] or orange [tdTomato, Fig. 2(c)] channels. Our selected combination of FPs, filters, and phantom optical properties yielded very similar SNRs for both GFP+ and tdTomato+ cells [Figs. 2(d) and 2(e)]—MM cells of both fluorophores had a mean SNR of 28.3 dB while the BC GFP+ and tdTomato+ cells had mean SNRs of 34.1 and 34.0 dB, respectively.

We also observed that some particularly bright individual cells and 1FP clusters result in 2λ peaks—one peak in the primary channel (green for GFP, orange for tdTomato) and one peak in the opposite channel due to fluorophore emission bleed [Fig. 2(f)]. However, we were able to distinguish these 1FP detections apart from detections of 2FP clusters as described in Sec. 2.2. To show this, we separately performed DiFC on suspensions of GFP+ and tdTomato+ MM cells (MM) and 1FP clusters (BC) in a phantom. We note that because MM cells are cultured in suspension they typically grow as individual cells and small clusters, whereas adherent 4T1 BC cells readily form large multicellular groupings in culture.^29^

**Fig. 3:**
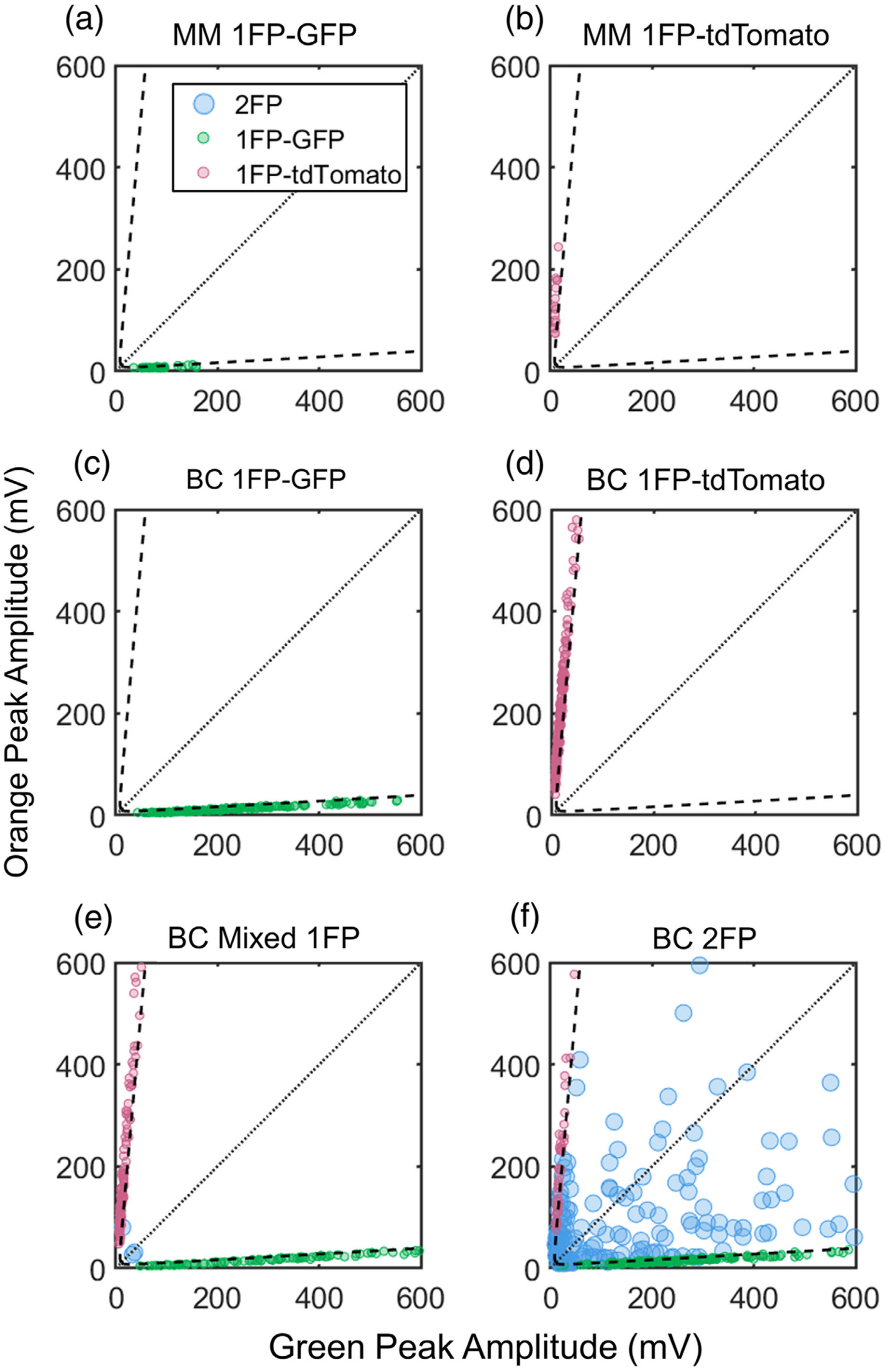
Green channel and orange channel peak amplitudes of 2λ peaks can be compared to identify 1FP and 2FP detections. The dotted diagonal lines indicate hypothetical 1:1 GFP and tdTomato detections, and the dashed curves represent Eq. (1), which is the threshold for 2λ peaks that distinguishes 1FP or 2FP detections. Therefore, points between the dashed lines indicate 2FP detections, and outside the lines indicate 1FP detections. (a,b) Bright MM single cells and small clusters detected with two-color DiFC were identified as GFP-only (a) or tdTomato-only (b). (c,d) GFP and tdTomato 1FP BC clusters were also detected as 1FP. (e) GFP and tdTomato 1FP BC clusters were mixed and scanned simultaneously which resulted in few false 2FP detections. (f) Co-cultured GFP and tdTomato BC cells and clusters resulted in many 2FP detections.

For all detected 2λ peaks, the green and orange peak amplitudes are plotted in Fig. 3. The dashed curves show the maximum expected amplitude of fluorophore spectral bleed between green and orange detection channels [Eq. (1)]. As such, detections plotted between the dashed lines are determined by our algorithm to be 2FP (blue circles) multi-cellular clusters and all detections outside the lines are 1FP (small green or pink circles) from single cells or 1FP clusters. The TR value in Eq. (1) was estimated as 0.057 for GFP+ cells and 0.089 for tdTomato+ cells by calculating the highest TR values of the single-fluorophore phantom MM and BC data with Eq. (2) [Figs. 3(a) and 3(b)]. We note that we selected a ‘conservative’ threshold that accounted for the worst-case coincidence of signal noise and peak detection. As shown in Figs. 3(a) and 3(b), this resulted in no false positive identification of 2FP clusters when running 1FP suspensions of cells through the phantom.

We also collected DiFC data of GFP+ clusters and tdTomato+ BC clusters through the phantom, either one FP at a time [Figs. 3(c) and 3(d)] or mixed in a combined suspension [Fig. 3(e)]. As shown, all detections were correctly identified as 1FP for the single FP cluster cases [Figs. 3(c) and 3(d)]. In the case of mixed 1FP cluster suspension [Fig. 3(e)], most detections were correctly identified as 1FP, although a small number were labeled as 2FP. This occurred for only a small proportion of detections (1.5%) during scans with high flow rates of 15.5 detections per minute. As such, we surmise that the errors were likely the result of coincident detections of both 1FP GFP+ and tdTomato+ clusters in the phantom (i.e., both passed through the ∼1  mm diameter DiFC field of view at the same time), as opposed to incorrect classification of a single detection.

Finally, we created 2FP clusters of BC CTCs by co-culturing GFP+ and tdTomato+ cells. Suspensions were passed through a phantom and scanned with two-color DiFC [Fig. 3(f)]. The resulting DiFC data showed both 1FP and 2FP detections, which were 53.9% and 46.1% of all 2λ peaks, respectively. There was a large variety of 2FP clusters, some with 50% GFP and 50% tdTomato and others with more GFP or tdTomato cells.

### 3.2 Two-Color DiFC in Multiple Myeloma Xenograft Model Mice *In Vivo*

**Fig. 4:**
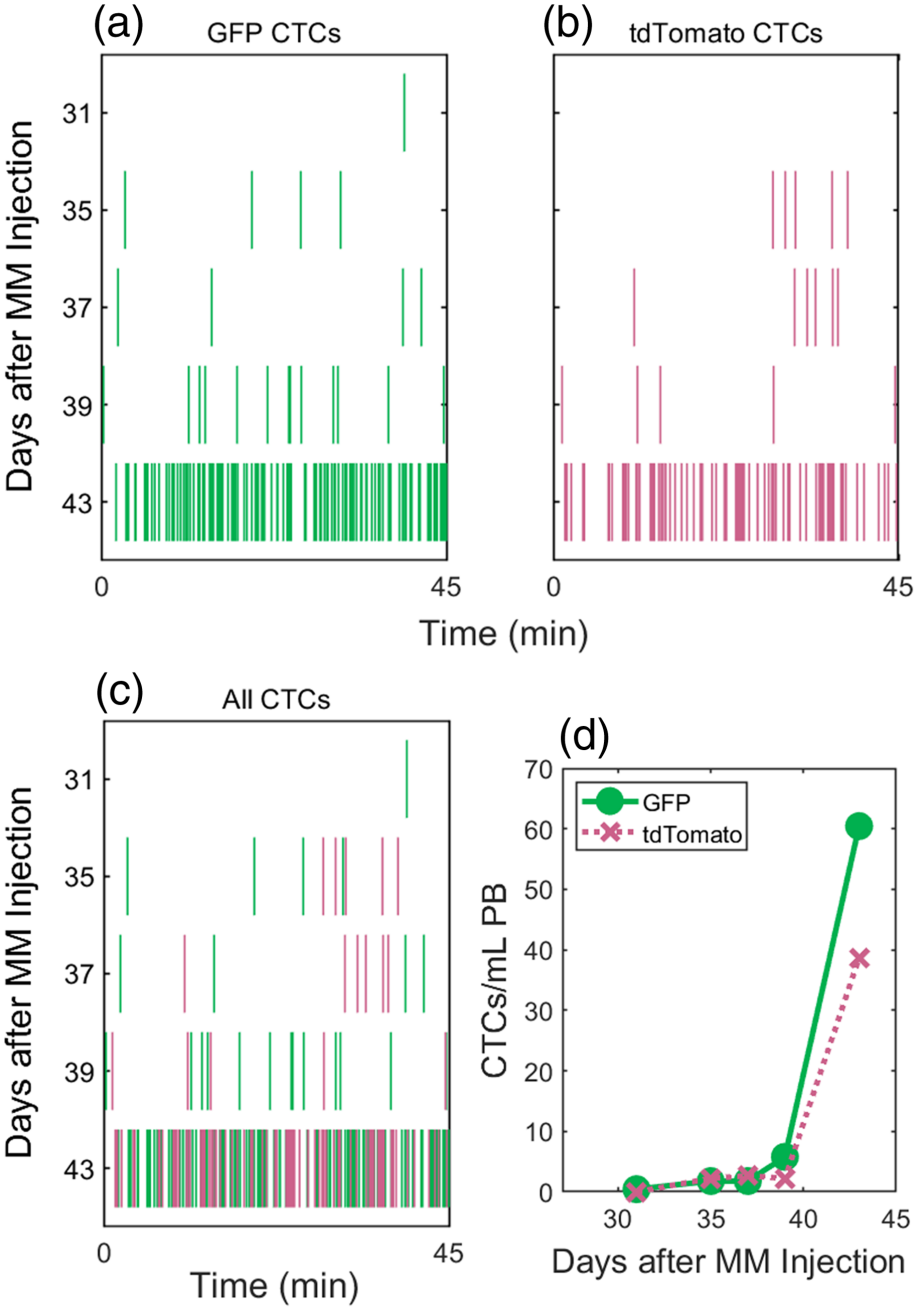
(a) GFP+ and (b) tdTomato+ MM CTC detections in a mouse over time in a disseminated xenograft model. Each vertical line represents a single CTC detection in time. (c) Both GFP and tdTomato detections are shown together. (d) The estimated concentration of CTCs in the peripheral blood (PB) increased over time and was similar for both cell populations.

We next performed two-color DiFC on MM tumor bearing mice [Fig. 4]. Mice were injected with a suspension of GFP+ and tdTomato+ MM cells (1:1 ratio). MM cells are known to initially rapidly home to the bone marrow niche after injection, steadily proliferate and then circulate in the peripheral blood in increasing numbers over time.^21^ Since cells were otherwise identical (aside from FP expression), we expected this proliferation would occur at approximately the same rate for both GFP+ and tdTomato+ cells. Representative two-color DiFC data measured periodically during tumor growth are shown in Figs. 4(a)–4(d). Here, Figs. 4(a) and 4(b) are raster plots, where each vertical line represents a DiFC detection of a GFP+ and tdTomato+ MM cell, respectively. When plotted together [Figs. 4(c) and 4(d)] we found that the number of both CTC types were, as expected, observed at approximately the same frequency and rate of increase.

### 3.3 Correlation of GFP and tdTomato CTCs

**Fig. 5:**
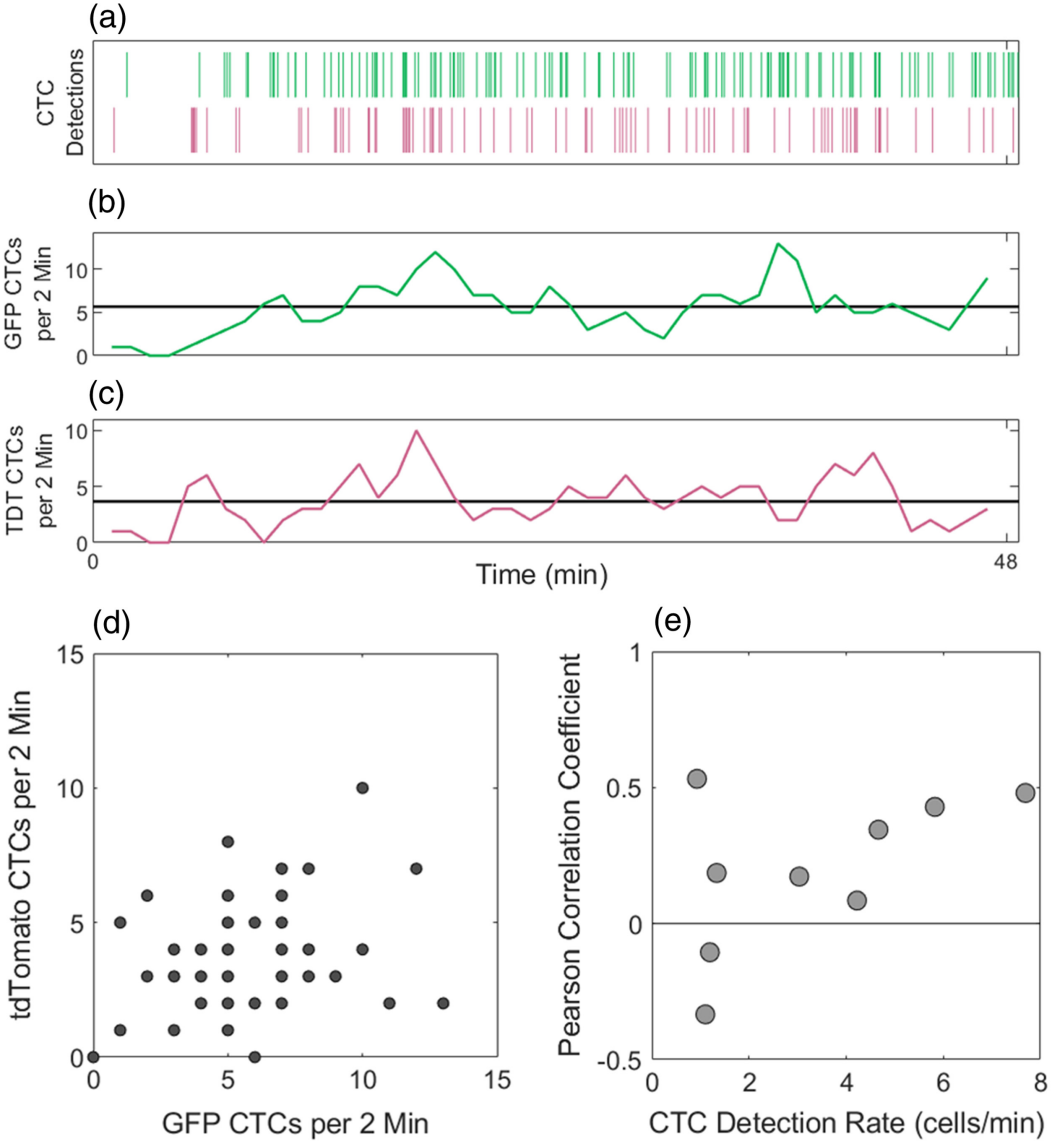
(a) Representative raster plots of GFP+ (first row) and tdTomato+ (second row) MM CTC detections from a DiFC scan. The numbers of (b) GFP+ and (c) tdTomato+ CTCs detected in moving 2-min intervals fluctuated over the length of the scan. (d) The Pearson correlation coefficient (PCC) of paired GFP and tdTomato detection rates was 0.287. (e) PCCs for all DiFC scans with at least 0.5 CTCs per minute are shown. Overall, these data indicate only weak correlation between GFP and tdTomato detection rates suggesting differences of CTC numbers in circulations as opposed to fluctuations in blood flow rates.

We also visualized DiFC CTC detections as moving averages, where CTCs are counted in 2-min moving intervals through the scan. Figure 5 shows a representative DiFC scan with individual cell detections [Fig. 5(a)] and moving averages through the scan for GFP [Fig. 5(b)] and tdTomato [Fig. 5(c)] detections. These data are typical of DiFC measurements,^11^ with transient periods of higher and lower detection rates observed during the scan for both GFP+ and tdTomato+ cells.

An open question was whether this variation in count rates is due to short-term variations in CTC shedding from tumor into circulation, or transient changes in cardiovascular output (and therefore DiFC blood volume sampling) while mice are under inhaled anesthesia. We investigated the correlation between the paired moving averages of GFP and tdTomato detections using the Pearson correlation coefficient (PCC) as shown in Fig. 5(d). For this scan, a PCC of 0.345 was obtained, suggesting only a weak correlation between the GFP and tdTomato detection rates (p-value of 0.018).

Likewise, we calculated the PCC for all DiFC scans in this study with overall detection rates of at least 0.5 CTCs detected per minute, as shown in Fig. 5(e). Overall, the PCC data showed weak positive correlation between the channels (median PCC of 0.186 and mean of 0.199). Since GFP and tdTomato detection rates are poorly correlated, this suggests that temporal fluctuations are due to transient changes in rates of CTC shedding into the bloodstream, as opposed to changes in vascular output.

### 3.4 CTC Clusters in Multiple Myeloma Mice

**Fig. 6:**
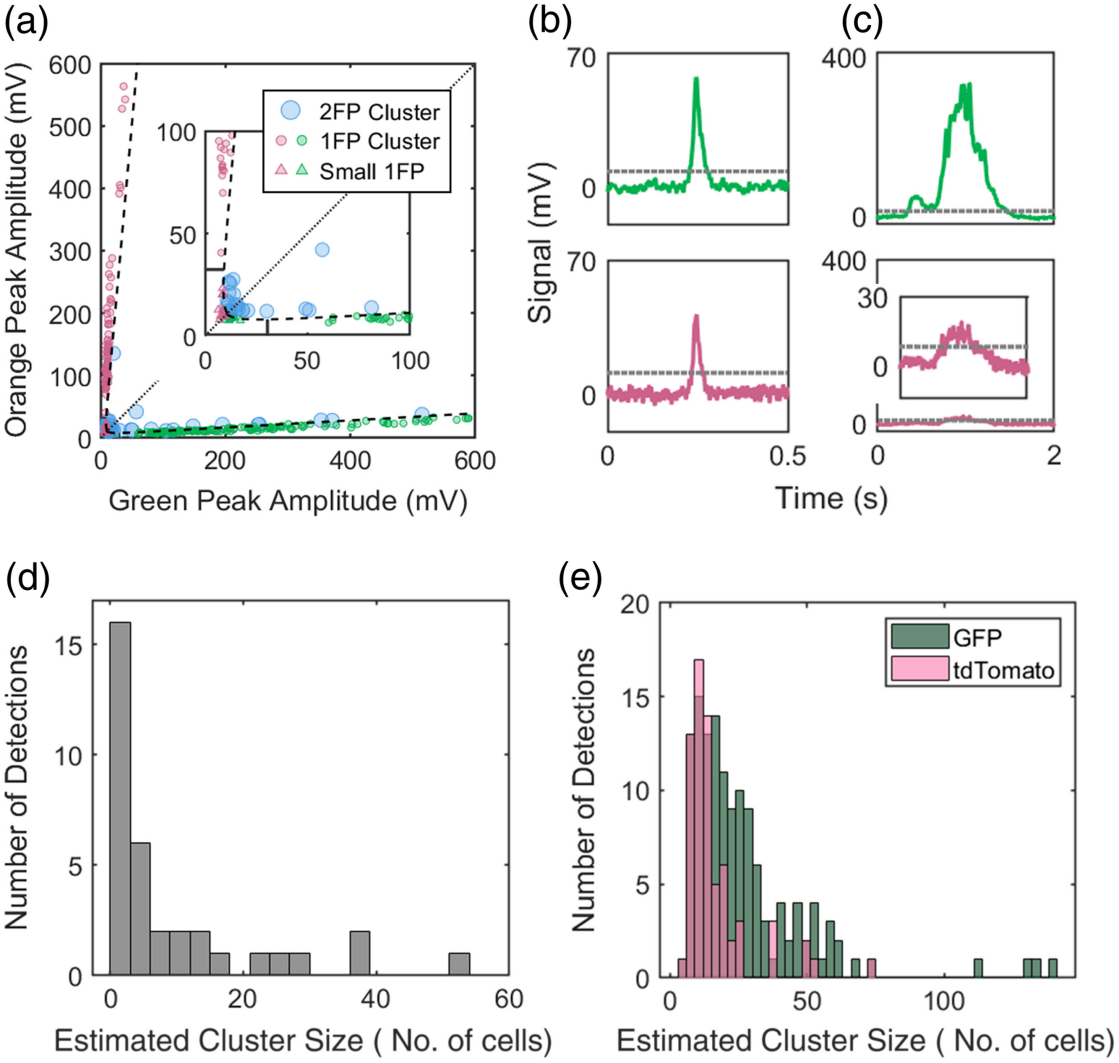
(a) Amplitudes of all 2λ peaks detected in MM mice. As in Fig. 3, the dashed curves indicate the threshold for which 2λ peaks are classified as 2FP or 1FP. The solid lines indicate the estimated amplitude of 3 GFP and 3 tdTomato CTCs. 1FP peaks with amplitudes of at least three CTCs are considered clusters. Representative (b) 2FP and (c) 1FP detections are shown. Histograms show the estimated sizes of (d) 2FP and (e) 1FP CTCCs (in number of cells).

A total of 260 2λ peaks were detected in the MM mice over a total 18 h of DiFC data. These are plotted in Fig. 6(a). Our analysis determined that 13.5% were 2FP detections [representative example is shown in Fig. 6(b)] and 86.5% were 1FP detections [representative example is shown in Fig. 6(c)].

We further estimated the size of the CTC clusters based on the combined peak amplitudes as summarized in Figs. 6(d) and 6(e). CTCCs are defined as two or more cells traveling together in circulation, or three or more nuclei travelling together in circulation. The latter definition excludes the possibility of a single CTC undergoing mitosis being identified as a CTCC.^5^ Therefore, in our calculations, 1FP detections that had amplitudes consistent with three or more GFP+ or tdTomato+ nuclei were considered 1FP clusters (89.3% of 1FP detections). 2FP detections contained at least one GFP- and tdTomato-expressing CTC, so that all 2FP detections were considered 2FP CTCCs. In combination, CTC clusters of either type (1FP or 2FP) represented 14.6% of all CTC detections. This is similar to the rate that we previously reported in a GFP-only MM tumor model.^21^

Figure 6(d) shows a histogram of cluster sizes for measured 2FP clusters (mean size = 9.3 cells; median size = 3 cells), and Fig. 6(e) shows a histogram of clusters sizes for measured 1FP clusters (mean size = 23.4 cells; median size = 16). The difference in estimated mean sizes here is due to the two (disparate) definitions used. However, in general this distribution of sizes is also similar to our previous work.^21^

We further note that we manually curated our CTCC detections after automated raw data processing and removed suspected artifacts, e.g., 2FP detections that appeared to arise from a motion artifact, or high amplitude (greater than 1500 mV) 2λ detections that saturated one of the detector PMTs and gave inaccurate estimation of the relative peak intensities.

### 3.5 Whole-Body Hyperspectral Fluorescence Cryo-Imaging

**Fig. 7:**
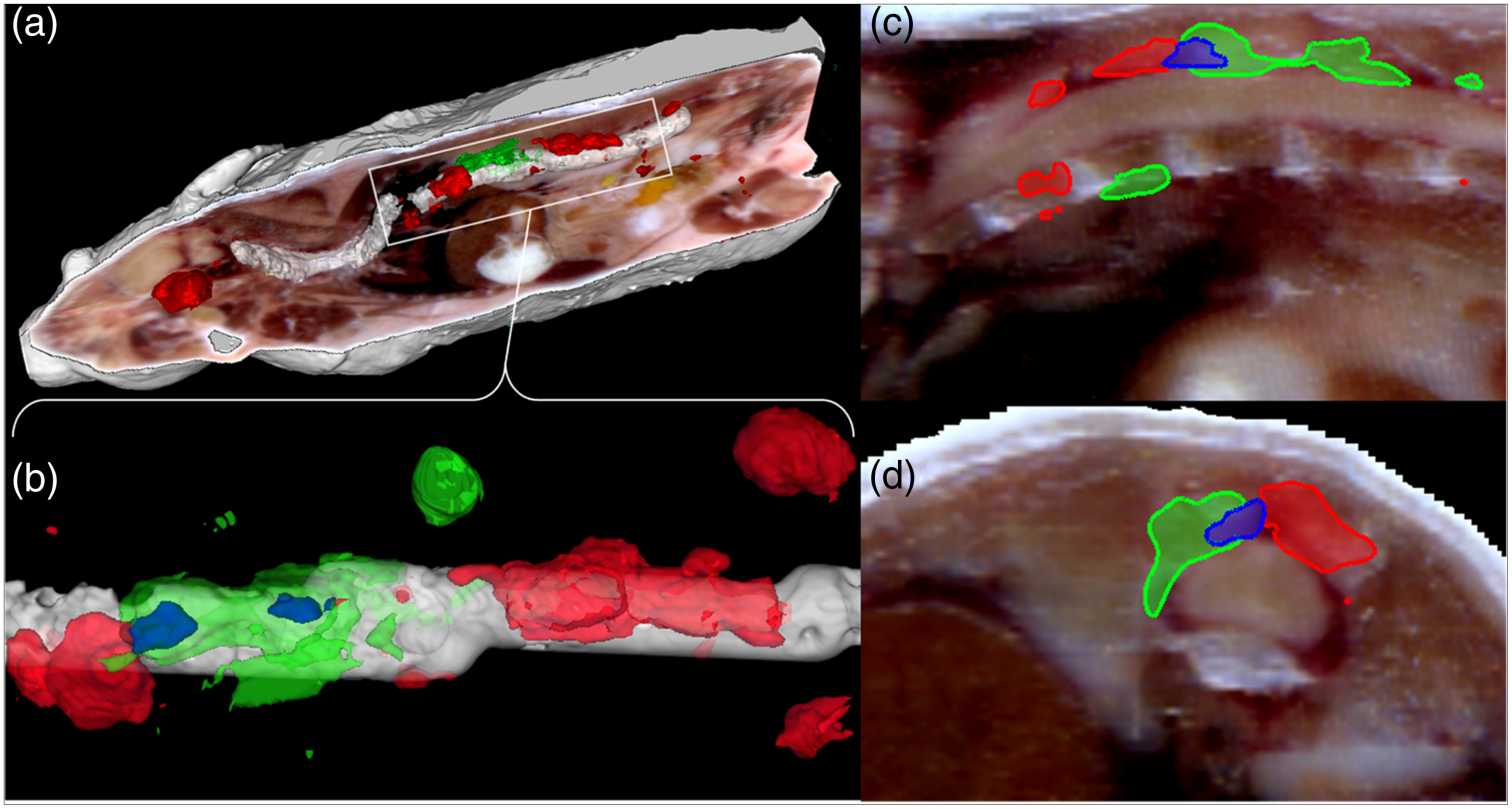
Hyperspectral fluorescence cryo-imaging showed that most tumor masses in the mice were single FP. This is consistent with our observation that >85% of CTCC detections were 1FP and not 2FP. (a) The full body image of a representative mouse is shown as well as (b) a top-down view of a section of the spine displaying two examples of 2FP tumor masses. (c) and (d) Additional 2-D cross-sectional sagittal and axial views of one 2FP tumor mass are also shown along the spine. GFP is shown in green, tdTomato in red, and overlap in blue.

After completion of DiFC scanning we performed whole-body hyperspectral fluorescence cryo-imaging of tumor bearing mice. Compared to whole body planar fluorescence or bioluminescence imaging techniques,^30^ cryo-imaging provides significantly superior spatial resolution and quantification of fluorescence signals.^27^ Based on prior characterization of the MM DXM model, we expected tumor to proliferate in the skeleton of the mouse.^31^ Hyperspectral fluorescence cryo-imaging confirmed these expectations, with GFP+ and tdTomato+ tumor appearing along and inside the spine, and within the brain, skull, scapula, humerus, femur, ribs and muscles of the legs, and arms. As shown in the whole-body rendering of a representative mouse [Fig. 7(a)] nearly all bulk tumor appeared to be single-FP, as opposed to homogenously mixed 2FP tumors. These data are consistent with our observation that most detected CTCCs were 1FP with relatively few 2FP CTCCs.

Upon close investigation of the spine in Fig. 7(b), two examples of mixed tumors can be seen in blue. One of the 2FP tumors is further visualized in sagittal and axial cross-sectional views along the spine in Figs. 7(c) and 7(d). Overall, though, mixed tumors represented only a small fraction of the total observed tumor volume.

## 4 Discussion and Conclusions

We previously developed several single-color DiFC systems that can non-invasively enumerate single-FP expressing CTCs to allow monitoring of metastatic dissemination in mice.^20^^–^^22^ In this work, we developed and validated a two-color DiFC system that allows us to simultaneously detect populations of GFP and tdTomato expressing CTCs. While multi-wavelength (multiplexed) intravital microscopy based methods of monitoring cells in blood flow have been reported previously,^32^^–^^34^ we have combined two-color detection with the advantages of DiFC which uses diffuse light to scan much larger volumes of blood than microscopy-based methods. Photoacoustic IVFC multiplexing has also been developed, which uses the photoacoustic effect to detect pigmented cells (such as melanoma) or cells labeled with absorbing contrast agents, such as carbon nanotubes.^15^

Detection of two similar CTC populations within the same mice allowed us to confirm that fluctuations in DiFC detections were unlikely due to changes in blood flow in the DiFC field of view (e.g., due to cardiovascular effects of the isoflurane anesthesia). The weak or moderate correlation between GFP+ and tdTomato+ detections therefore supports the notion that observed fluctuations in CTC numbers were caused by rates of CTC shedding from tumors and rapid clearance from circulation.^11^^,^^35^

Additionally, two-color DiFC allowed for detection of CTC clusters in cases where CTCCs contain cells expressing both FPs. The mouse tumor model studied here had evidence of some 2FP clusters; however, most CTCC detections were either GFP+ or tdTomato+ only. Post-mortem whole-body hyperspectral fluorescence cryo-imaging showed that the vast majority of the MM tumor volume in and adjacent to the bone expressed a single FP. Small regions of overlap [e.g., Figs. 7(c) and 7(d)] were observed, although the apparent mixing may be at least partly due to the resolution limits of the cryomacrotome system. Since we injected a mixed suspension of GFP+ and tdTomato+ MM cells at the time of inoculation, these data suggest that initial tumors were formed by single MM cells that homed to bone marrow and proliferated into tumor masses, as opposed to groups of MM cells forming masses. Therefore, there were few tumor masses composed of a mixture of GFP and tdTomato expressing cancer cells. This is consistent with the prevalence of 1FP CTCCs in our DiFC measurements and rarity of 2FP CTCCs. This is also consistent with prior work in breast cancer bearing mice which showed that mice implanted with two, single-FP expressing tumors in bilateral breast pads formed metastases that also primarily expressed single FPs.^3^ We note that the data suggests that while initial tumors were formed by single MM tumor cells, it is possible (or likely) that subsequent proliferation through the bloodstream may have been facilitated by shedding of CTCCs, which are known to have higher metastatic potential.^3^^–^^5^

We also noted that false positive 2FP detections could occur due to rare motion artifacts or when two CTCs of different FPs flow past the DiFC probes at similar times. Here, these cases were identified by manual operator inspection. In the future, more rigorous signal processing and pattern recognition methods could be used to better discriminate 2FP CTC clusters. Improved processing will facilitate further experiments with solid tumor models with more abundant 2FP clusters.

In summary, two-color DiFC facilitates a large range of experiments in which two populations of cells can be studied. For instance, anti-cluster therapies could be studied longitudinally.^36^ We could also observe the CTC shedding patterns of two subpopulations of cancer cells in the same tumor or in two tumors. This could allow study of a therapeutic’s effects on treatment-resistant and -responsive cancer cells within the same mouse, reducing inter-mouse variability when studying these tumors in separate mice.^37^ Additionally, two completely different cell types could be detected, such as CTCs and tumor-associated macrophages to observe how the latter influences the development of metastases.^38^ Although the focus of the present study was development and validation of a small animal pre-clinical research system, in our lab we are also studying potential clinical translation of DiFC through the use of molecularly-targeted contrast agents.^22^^,^^39^^,^^40^

## Supplementary Material



## Data Availability

The data presented in this article are publicly available in the Pennsieve repository at DOI: 10.26275/q2w0-keol. The archived version of the code described in this article can be freely accessed through the GitHub repository at https://github.com/mark-niedre/Two-ColorDiFC
