# Supplementary material for: Two-color diffuse in vivo flow cytometer
Source: J Biomed Opt. 2024 May 30;29(6):065003. doi: 10.1117/1.JBO.29.6.065003 (PMC11138342; doi:10.1117/1.JBO.29.6.065003)
Supplement: Supplementary file 1 [file JBO_029_065003_SD001.pdf]

Supplemental Material

As discussed in the main body of the paper, "2λ peak detections", i.e. detection of simultaneous peaks in both green and orange channels on a single probe may be generated by both two-color CTCCs and single-color CTCCs due to spectral bleed between the fluorescence channels. To distinguish between these events, we examined the relative amplitudes of the two peaks. The larger of the two peaks is considered the primary peak and the smaller peak is considered the secondary peak. The ratio of the secondary peak amplitude (I_{sec}) to the primary peak amplitude (I_{pr}) is expected be larger for two-color detections than single-color.

In a noiseless system, single-color detections would result in consistent ratio of the 2λ peak amplitudes ($I'_{sec}\ /I'_{pr}$). We refer to this as the true ratio (TR). However, random measurement noise on each channel (ε_1 for the secondary peak and ε_2 for the primary peak) from electronic or physiological sources affects the detected amplitudes and resulting ratio as follows:

$$\frac{I_{sec}}{I_{pr}} = \frac{I'_{sec},+\varepsilon_1}{I'_{pr}+\varepsilon_2}\,. \tag{S1}$$

Because we will define two-color detections as 2λ peaks with amplitude ratios larger than single-color detections, we calculate the largest ratios that may result from random noise (Eq. S2), which we assume is the case where the secondary peak occurs with the highest possible noise ($\max(\varepsilon_1)$) and the primary peak coincides with the lowest possible noise ($\min(\varepsilon_2)$, which is negative).

$$\max\left(\frac{I_{sec}}{I_{pr}}\right) = \begin{cases} \frac{I'_{sec}+\max(\varepsilon_1)}{I'_{pr}+\min(\varepsilon_2)}, & I'_{pr} \geq I'_{sec} + \max(\varepsilon_1) - \min(\varepsilon_2) \\ \frac{I'_{pr}+\min(\varepsilon_2)}{I'_{sec}+\max(\varepsilon_1)}, & else \end{cases} \tag{S2}$$

In the first case, $I'_{pr} \geq I'_{sec} + \max(\varepsilon_1) - \min(\varepsilon_2)$, we have

$$\frac{I'_{sec}+\max(\varepsilon_1)}{I'_{pr}+\min(\varepsilon_2)}=\frac{\frac{I'_{sec}}{I'_{pr}}*I'_{pr}+\max(\varepsilon_1)}{I'_{pr}+\min(\varepsilon_2)} \tag{S3}$$

$$=\frac{TR*I'_{pr}+\max(\varepsilon_1)}{I'_{pr}+\min(\varepsilon_2)}. \tag{S4}$$

Our experimentally collected two-color DiFC data shows that in general noise ranges between [-5, 5] mV, so

$$\frac{I'_{sec}+\max(\varepsilon_1)}{I'_{pr}+\min(\varepsilon_2)}=\frac{TR*I'_{pr}+5}{I'_{pr}-5}. \tag{S5}$$

We therefore estimate the maximum threshold for a single-color detection ratio as:

$$\max\left(\frac{I_{sec}}{I_{pr}}\right)\approx\frac{TR*I_{pr}+5}{I_{pr}-5}. \tag{S6}$$

We further estimate TR as the largest value measured from DiFC detections in an optical phantom (primary peak amplitude I^*_{pr}, secondary peak amplitude I^*_{sec}) with known suspensions of single-color CTCCs. This was calculated separately for GFP and tdTomato CTCC suspensions where the primary peaks were either green or orange, respectively.

$$TR\approx\frac{\frac{I^*_{sec}}{I^*_{pr}}(I^*_{pr}-5)-5}{I^*_{pr}} \tag{S7}$$

We then use Eq. S6 and Eq. S7 to identify 2λ as single-color or two-color in Sec. 2.2.

Finally, it is hypothetically possible that noise could make the secondary peak appear larger than the primary peak (as in the second case in Eq. S2). In this situation, the same analysis as above and in Sec. 2.2 would apply with the primary and secondary peaks in a noiseless system observed as the secondary and primary peaks respectively.
